# Relating neurological symptoms to cerebral metastases at the time of initial staging scan

**DOI:** 10.1186/1470-7330-15-S1-P17

**Published:** 2015-10-02

**Authors:** C Corbally, S Bready, O Cram

**Affiliations:** 1 New Southern General, Greater Glasgow and Clyde NHS, Glasgow, UK

## Aims

Cerebral metastases represent a significant obstacle in the treatment of patients with systemic malignancies, occurring in 10-30% of patients with cancer. Brain imaging in neuro-asymptomatic patients is advised for some malignancies but not for others. Our aim was to examine the incidence of cerebral metastases in patients with a new diagnosis of the primary malignancies that commonly spread to the brain, and relate this with the patients’ neurological symptoms, if any.

## Methods

Using our PACS and reporting system we retrospectively looked at all initial staging scans performed in the year 2013 for the following primaries: lung, breast, colorectal, melanoma, renal. From this list we selected out those patient in whom a CT brain was performed within six weeks of staging to assess for metastases. Using the hospital referral system and electronic records, we compared this with the patients’ neurological symptoms at time of scanning.

## Results

From a total of 4063, 343 patients met our inclusion criteria, the vast majority of which were patients with a lung primary (266). We found varying rates of positive scans among the different primary malignancies, approaching 44% for lung primaries. While the most common indication for brain imaging was altered mental state, the symptom most predictive of cerebral metastases was new seizure activity.

## Conclusion

Our findings support routine brain imaging for patients with lung primaries, in keeping with the literature. Although it was the most common indication, altered mental state such as confusion was not very predictive of cerebral metastases.

